# Noise propagation through extracellular signaling leads to fluctuations in gene expression

**DOI:** 10.1186/1752-0509-7-94

**Published:** 2013-09-25

**Authors:** Omar P Tabbaa, German Nudelman, Stuart C Sealfon, Fernand Hayot, Ciriyam Jayaprakash

**Affiliations:** 1 Department of Physics, Ohio State University, Columbus 43210, USA; 2 Department of Neurology, Mount Sinai School of Medicine, New York 10029, USA; 3 Center for Translational Systems Biology, Mount Sinai School of Medicine, New York 10029, USA

**Keywords:** Cytokine signaling, Dendritic cells, Multi-scale modeling, Noise propagation, Spatial heterogeneity

## Abstract

**Background:**

Cell-to-cell variability in mRNA and proteins has been observed in many biological systems, including the human innate immune response to viral infection. Most of these studies have focused on variability that arises from (a) intrinsic stochastic fluctuations in gene expression and (b) extrinsic sources (e.g. fluctuations in transcription factors). The main focus of our study is the effect of extracellular signaling on enhancing intrinsic stochastic fluctuations. As a new source of noise, the communication between cells with fluctuating numbers of components has received little attention. We use agent-based modeling to study this contribution to noise in a system of human dendritic cells responding to viral infection.

**Results:**

Our results, validated by single-cell experiments, show that in the transient state cell-to-cell variability in an interferon-stimulated gene (*DDX58*) arises from the interplay between the spatial randomness of the cellular sources of the interferon and the temporal stochasticity of its own production. The numerical simulations give insight into the time scales on which autocrine and paracrine signaling act in a heterogeneous population of dendritic cells upon viral infection. We study the effect of different factors that influence the magnitude of the cell-to-cell-variability of the induced gene, including the cell density, multiplicity of infection, and the time scale over which the cellular sources begin producing the cytokine.

**Conclusions:**

We propose a mechanism of noise propagation through extracellular communication and establish conditions under which the mechanism is operative. The cellular stochasticity of gene induction, which we investigate, is not limited to the specific interferon-induced gene we have studied; a broad distribution of copy numbers across cells is to be expected for other interferon-stimulated genes. This can lead to functional consequences for the system-level response to a viral challenge.

## Background

Cell-to-cell variability in the expression levels of mRNA and proteins has been studied extensively [1-8]. This variability has been shown to arise (a) from the intrinsic stochastic fluctuations in biochemical reactions such as those that regulate the expression of genes [2] as well as (b) from extrinsic sources such as cell-to-cell fluctuations of the number of limiting transcription factors or (c) from extracellular, environmental diversity. A variety of functional roles have been suggested for cellular noise [9]: the heterogeneity in expression levels can determine phenotypic behavior of the cell [6] or affect the timing of events and responses to stimuli. It has been suggested that the noisiness of human dendritic cells’ interferon response to infection may help avoid a cytokine storm [10]. Also, heterogeneity in the numbers of Tat proteins has been suggested as an explanation for the latency of HIV [11].

In the field of gene expression noise, both in experimental investigations and theoretical analyses, most of the attention has been restricted to intracellular noise in simple systems, such as genetic circuits [12,13] or a connected set of cellular reactions [14]. Noise has been studied experimentally in a variety of cells, ranging from bacteria [3,15] to mammalian cells [10,16]. A significant contribution [17] was the distinction between two sources of noise, intrinsic (due to stochasticity of the underlying biochemical process) and extrinsic (due to variability in cellular components that affect gene expression), which could be separately measured. In contrast, in many situations, cells communicate with each other through extracellular signaling and do not act independently. In this theoretical study, we study spatially-distributed sources of induced gene noise arising from extracellular communication through paracrine and autocrine signaling. The focus is therefore not on single cells evolving independently, but on a collection of cells that interact with each other to create cell-to-cell variability in the expression levels of induced genes. Our investigation is motivated by our previous experimental work on the innate immune response of dendritic cells (DCs) to viral infection, which showed large cell-to-cell variability of IFN-β mRNA [10] as well as of DDX58 [18], a gene induced as a result of interferon secretion and cognate receptor binding. The question arises whether the broad, non-Poisson experimental distribution of DDX58 is related to the temporally sporadic IFN-β production. At first glance this seems unlikely, because in the extracellular medium, homogenization of secreted cytokines is expected to occur. This is indeed the case, but can happen many hours after viral infection. Our work shows that there is a period of approximately 10 hours where the spatial heterogeneity of infection (only a fraction of cells is infected) and the temporal stochasticity of interferon induction create an environment where genes induced by interferon signaling are very noisy as well.

For our simulations, we use a recently developed multi-scale, stochastic simulation method [18,19] coupling extracellular diffusion with intracellular reactions for many cells. We use a simplified model to account for the various intracellular processes and to demonstrate the possibility of robust early activation of a subset of cells in agreement with experimental results. In this earlier agent-based model, the excluded elements were: the noise due to the enhanceosome assembly in interferon induction and the impact of transcriptional noise in the induced gene (due to bursting) on cell-to-cell variability. This is incorporated explicitly in a more detailed model that focuses on the way intra-cellular noise in cytokine production is transmitted and re-shaped by a cascade of processes, including spatial diffusion and noisy downstream intracellular reactions. Our quantitative description allows us to identify the time scales over which different aspects of cell-to-cell variability occur. Furthermore, the mechanistic understanding of the different processes makes it possible to predict the effect of changing experimental conditions such as the density of cells, the level of infection, or the varying rates of biochemical reactions for different viruses. Our proposed mechanism leads to the prediction that under the conditions posited in the simulation, similar broad distributions should occur for other stimulated genes in infectious and similar biological situations.

## Results

We describe the results of our simulation of the effect of extracellular signaling on noise with reference to the experimental measurements in Newcastle disease virus (NDV) infected DCs [18,20]. Comparison with experiment helps in setting model parameter values and the time scales over which cell-to-cell variability develops. There is substantial evidence that cell-to-cell heterogeneity is cellular in origin [18,21-23]. It is the stochastic production of the IFN-β mRNA, observed experimentally and reproduced *in silico,* that drives the proposed extracellular mechanism and is the source of the broad distribution of interferon-induced genes (such as *DDX58*) through autocrine and paracrine signaling. We demonstrate the spatially-induced character of the observed noise, demonstrate its dependence on cellular rate constants, and present results for how cell-to-cell variability progresses from initial intracellular noise through extracellular fluctuations to intracellular noise induced by the latter.

### Spatial heterogeneity of extracellular cytokine density decreases as time evolves

**Figure 1:**
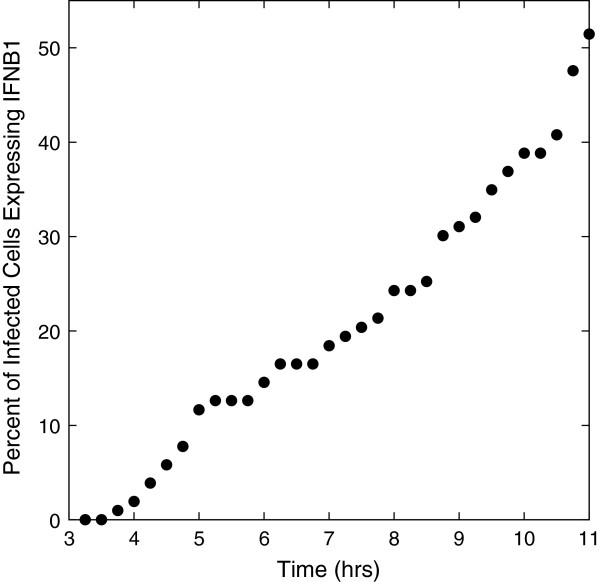
**The percent of infected cells (only infected cells can express the *IFNB1 *** **gene) with IFN-β mRNA vs. hours post-infection.**

**Figure 2:**
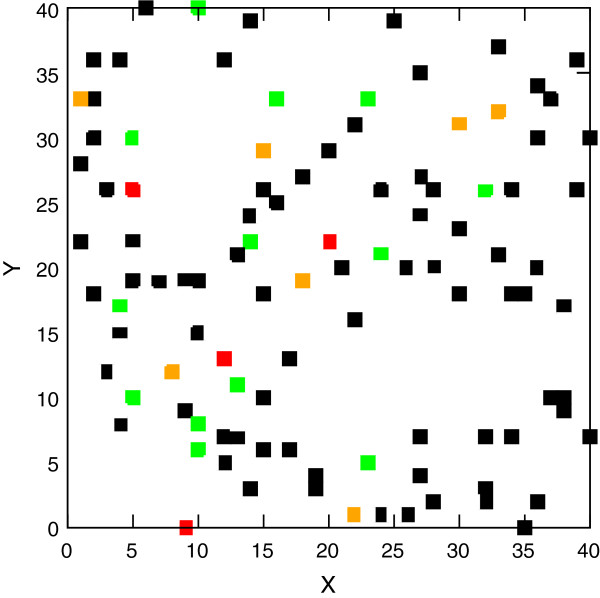
**Spatial distribution of IFN-β mRNA copy numbers in infected cells at 11 hours.** The numbers are color-coded with black denoting an IFN-β mRNA number less than 375, green a number between 375 and 750, orange between 750 and 1125, and red denoting numbers larger than 1125. The X- and Y-axes are in units of single cell length (~30 μm).

The initial response to viral infection leads to population-wide interferon induction that is spatially and temporally heterogeneous. The subsequent export of the cytokine gives rise to a spatially heterogeneous cytokine density profile. Interferon induction in infected cells is stochastic and shows considerable temporal and spatial heterogeneity, as demonstrated in Figures 1 and 2 respectively.

Only infected calls can express IFN-β and at any given time only a fraction of the infected cells have a fully assembled enhanceosome required for IFN-β induction. We determine the fraction of infected cells with nonzero IFN-β mRNA number. Figure 1 shows the fraction of infected cells that contain IFN-β mRNA. It takes 3 to 4 hours before some infected cells produce IFN-β mRNA. Thereafter, the fraction of infected cells expressing interferon rises, with a doubling of percentage from 6 to 9 hours, reaching 50% by 11 hours. The slow increase demonstrates a controlled response of the cell population to viral infection. The numbers are illustrative and depend on the rate constants of the model; they are consistent with the low fractions of infected murine fibroblast cells expressing *IFNB1*[22]. The time scales of our simulations differ since our rate constants were chosen to fit the data for human monocyte-derived DCs.

The stochastic assembly of the IFN-β enhanceosome and the bursting production [24,25] lead to temporal fluctuations in IFN-β mRNA number and spatial heterogeneity at all simulation times. As an example we show in Figure 2 the spatial distribution of cells at 11 hours (labeled in color to represent their number of IFN-β transcripts). As described in the figure caption, the copy numbers range from small (black squares) to large (red squares), through intermediate values (green and orange squares). The colored square distribution is quite heterogeneous, though at 11 hours it is less so than at earlier times where fewer cells contain IFN-β mRNA (see Figure 1). While the IFN-β mRNA distribution is spatially heterogeneous at all simulation times, the IFN-β protein distribution conversely becomes spatially homogeneous at later times (approximately 11 hours post-infection), as described below.

**Figure 3:**
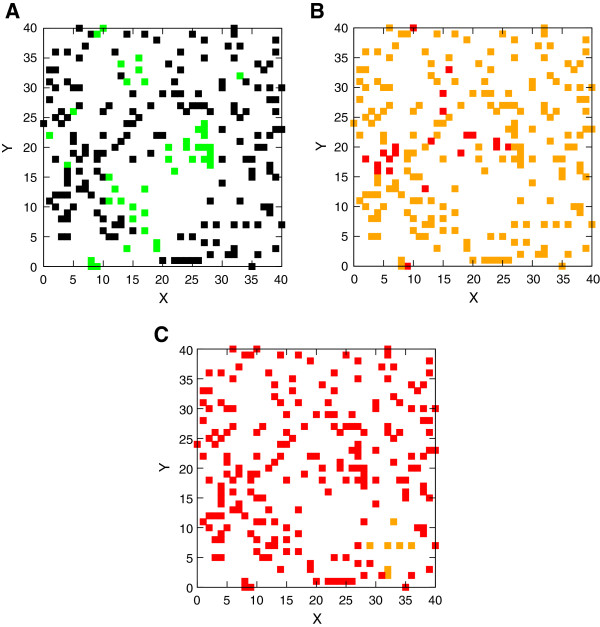
**Spatial distribution of the number of IFN-β surrounding dendritic cells.** Displayed are the distributions at **(A)** 6 hours **(B)** 9 hours and **(C)** 11 hours post-infection. The colors indicate the range of the number of cytokines in each box surrounding a DC: black denotes an IFN-β protein number less than 133, green a number between 133 and 400, orange a number between 400 and 1200, and red a number greater than 1200. More than 80% have fewer than 133 at 6 hours while 97% have more than 1200 cytokines at 11 hours showing the emergence of spatial homogenization. The X- and Y-axes are in units of single cell length (~30 μm) as in Figure 2.

The spatially heterogeneous export of IFN-β protein from different infected cells with the *IFNB1* gene activated at different times leads to a diffusion problem with sources that are distributed non-uniformly in space and turned on over a range of times. The secreted cytokine diffuses in the extracellular medium; this is a relatively fast process with a diffusion constant of 10 μm^2^ s^-1^ (see Methods) and it takes less than one minute for a secreted cytokine to diffuse to a nearby cell on the average. We study the time evolution of the spatial cytokine density profile. The inhomogeneous sources lead to an initially spatially inhomogeneous distribution of the cytokine that becomes more homogeneous as time progresses. This is displayed in Figure 3. Each square shows the location of a DC; we have color-coded the number of unbound IFN-β protein in its vicinity (only IFN-β within a square box containing a cell can bind to a free Interferon-α/β receptor (IFNAR) on the cell membrane). More than 80% of cells have a small number of cytokines (black squares, Figure 3A) in the same box at 6 hours post-infection because the *IFNB1* gene has not been turned on in most infected cells; this number declines to zero by 9 hours (Figure 3B). By 11 hours 97% of the boxes contain many cytokines (red squares, Figure 3C). Following the initial secretion of IFN-β it takes 3 to 5 hours for the homogenization of the cytokine density. The homogenization of the cytokine density on the scale of a few hours allows the system to develop a more coherent response to viral infection, despite the large stochasticity in the induction of the key sentinel molecule IFN-β [10,22].

### Effect of autocrine and paracrine signaling on the spatio-temporal distribution of bound interferon receptors

A broad spatial distribution of the fraction of bound IFNAR arises due to the spatio-temporal distribution of secreted IFN-β, and is enhanced by early autocrine effects. We present results for the distribution of the number of bound IFNAR across the cells as a function of time; we divide the cells into an activated group consisting of infected cells with nonzero IFN-β expression and another group consisting of both uninfected cells and infected cells with zero IFN-β expression. In the former group, the bound receptors arise from a combination of autocrine and paracrine signaling while for the latter group, only paracrine signaling is present. We are able to evaluate the relative importance of autocrine and paracrine signaling.

**Figure 4:**
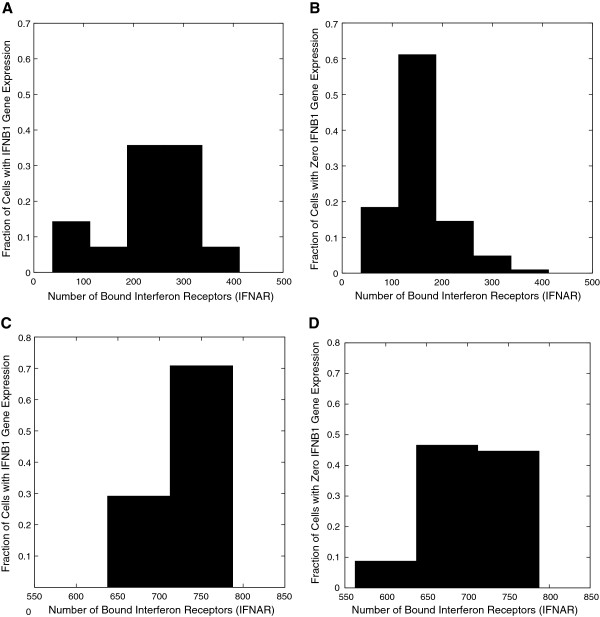
**Distribution of bound interferon receptors (IFNAR) in cells with and without IFN-β mRNA. (A)** For cells containing IFN-β mRNA and **(B)** for cells with no IFN-β mRNA plotted at 6 hours. The larger fraction above 200 in **(A)** indicates the importance of autocrine signaling at early times. The distribution of bound IFNAR at 11 hours post-infection is displayed in **(C)** for cells with and in **(D)** for cells without IFN-β mRNA.

In Figure 4 we display a histogram of a fraction of cells that correspond to a specified range of bound IFNAR numbers. Figures 4(A) and (B) correspond respectively to the distributions at 6 hours for cells with and without IFN-β mRNA. While the number of activated cells is much smaller at early times, a large fraction shows bound receptor numbers above 200. In contrast, very few cells without IFN-β mRNA have as many bound receptors. This clearly demonstrates the significant effect of autocrine signaling at early times (4 – 6 hours post-infection). By 11 hours (see Figures 4(C) and (D)) both the activated and inactivated cells distributions are essentially the same, showing the increasing influence of paracrine signaling as more infected cells are activated, an effect that underlies homogenization.

We quantify the greater importance of autocrine signaling at times immediately after interferon induction and illustrate this in Additional file 1: Figure S1. At around 4 hours post-infection (which is approximately the first time interferon induction begins in a small fraction of the infected cells) we find that the positive feedback from autocrine signaling increases the IFN-β production in those cells, thus magnifying the heterogeneity. The early autocrine signaling enhances the production of the interferon cytokine in early responders (the cells that produce IFN-β earliest) causing a decrease in the probability for attachment of the IFN-β protein to IFNAR receptors on the early responders, which leads to increased paracrine signaling. At approximately 6 hours post-infection the paracrine loop begins to prime the uninfected cells (the fraction of bound receptors on them increases sufficiently to robustly activate internal signaling cascades). For times greater than around 8 hours post-infection, paracrine signaling dominates and the system starts to respond coherently to the viral infection.

The bound IFNAR on both infected and uninfected cells activate the JAK/STAT pathway, leading to the induction of interferon-induced genes [26-28]. We choose rate constants such that the DDX58 production rate reaches half maximum when the number of bound receptors is 475 out of a total 1000. We study the spatial distribution of bound IFANR as a function of time in our *in silico* experiments. In Additional file 1: Figure S2 the distribution is shown as a spatial heat plot at different times. This follows the behavior of the homogenization of the IFN-β cytokine profile. These results demonstrate the importance of autocrine signaling at early times (4 – 6 hours post-infection, which enhances the spatial heterogeneity of bound receptors) and the importance of paracrine signaling in quenching the spatial heterogeneity at later times (> 8 hours post-infection).

### Large cell-to-cell variability of interferon-induced gene

IFNAR bound by IFN-β phosphorylate the STAT proteins that associate with IRF9 to form the pleiotropic transcription factor ISGF3. ISGF3 binds to the interferon-stimulated response element (ISRE) of many inducible genes. We choose to model *DDX58* that codes for RIG-I as an example of an interferon-induced gene since it has been studied experimentally [18] and shows a very broad distribution of mRNA number at 11 hours. We incorporate into our simulation the induction of the *DDX58* gene with a simple, coarse-grained model. The gene has two states, one with a basal production level in the absence of ISGF3, and one with an activated production rate with the transition between the gene states occurring at a rate proportional to (B/475)^2^ where B is the number of bound receptors (the value 475 corresponds to the threshold value of bound receptors). The cell-to-cell variability in DDX58 number arises from the promoter switching between states with basal and enhanced transcription rates. The rate of switching depends indirectly on the number of bound receptors and is itself time-dependent.

**Figure 5:**
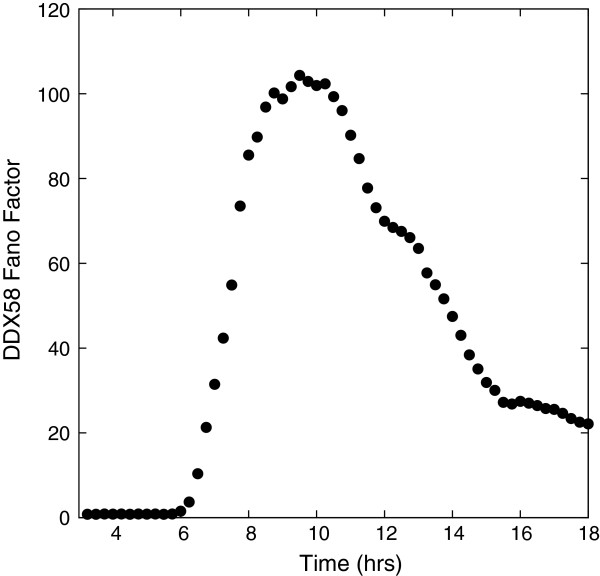
**Time evolution of the DDX58 Fano factor averaged across the cell population.** The Fano factor increases rapidly in a span of less than two hours and after reaching a plateau it decreases much more gradually.

This stochastic switching between a low (basal) and enhanced (promoted by ISGF3) production rate of DDX58 leads to bursts of high and low mRNA production and thus to a broad distribution of DDX58 mRNA, in agreement with experimental results. This behavior is also reflected in the distribution of the protein RIG-I that it encodes, which is displayed in Additional file 1: Figure S3. A simple quantification of the mRNA distribution is obtained by calculating the Fano factor (= Variance/Mean) averaged over all cells as a function of time. Our results are shown in Figure 5. In the absence of infection, the steady-state number of DDX58 follows a Poisson distribution with a Fano factor of 1. After infection, once the cytokines are released, the DDX58 gene can make a transition to the state with the higher induction rate. The Fano factor of the DDX58 mRNA distribution increases rapidly from unity in the basal state to values larger than 50. Interferon induction starts around 4 hours post-infection, as can be seen in Figure 1. The signaling is slightly biased towards paracrine signaling by seven hours post-infection (roughly three hours after interferon induction starts), as is evident from Additional file 1: Figure S1; by this time the Fano factor exceeds 50. After reaching a plateau, the Fano factor declines gradually, as most of the cells have activated JAK/STAT pathways, and the *DDX58* gene approaches a steady state, toggling between the two states of induction. The experimentally-observed Fano factor at 11 hours is around 100, in agreement with our *in silico* study. As to the 6-hour experimental data, given the small number of cells, the statistics are insufficient to make a precise comparison.

The Fano factor increases in Figure 5 in a time interval when the majority of cells have the number of bound receptors in a range around the threshold value. The effect of the bound receptors on the average rate of *DDX58* induction is shown in Additional file 1: Figure S4A. The effect of spatial inhomogeneity in bound IFNAR manifests itself most strongly when the majority of DCs have bound receptors in a range (200 to 600) around the threshold value of 475. In Additional file 1: Figure S4B we plot the fraction of DCs with bound IFNAR in the specified range, showing that it has a broad distribution. This corresponds to the range of time in Figure 5 over which the Fano factor for DDX58 is increasing.

**Figure 6:**
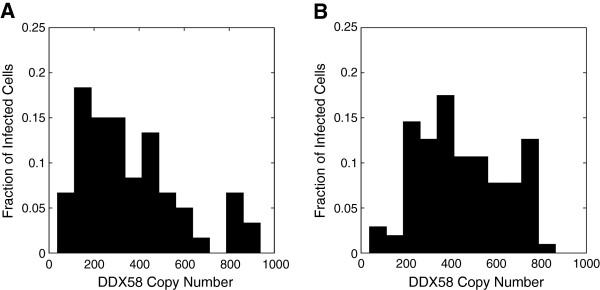
**Comparison of the experimentally measured DDX58 copy number distribution with simulations.** The experimental distribution at **(A)** 11 hours post-infection is obtained from Hu et al. [18], which consists of 200 single cell measurements. The comparison with the distribution from the simulation at **(B)** 11 hours post-infection shows good agreement.

In addition to the Fano factor, which is a single number characterizing the non-Poisson nature of the DDX58 distribution across cells, we study the full distribution. Experimentally, a surprisingly broad DDX58 distribution across the cell population is found at 11 hours post-infection [18]. We compare the DDX58 distribution obtained in our model with the published experimental results. We re-plot the data in Figure 4B of [18] as a histogram in Figure 6A at 11 hours. The simulation results at 11 hours are plotted in Figure 6B. The agreement is good considering the fact that there are only 200 cells in the experiment, and provides support to our hypothesis that cell-to-cell variability in IFN-β gene induction leads to large cell-to-cell variability in DDX58. This validates our proposed mechanism of intracellular stochasticity leading (via extracellular signaling) to noise in induced gene expression.

**Figure 7:**
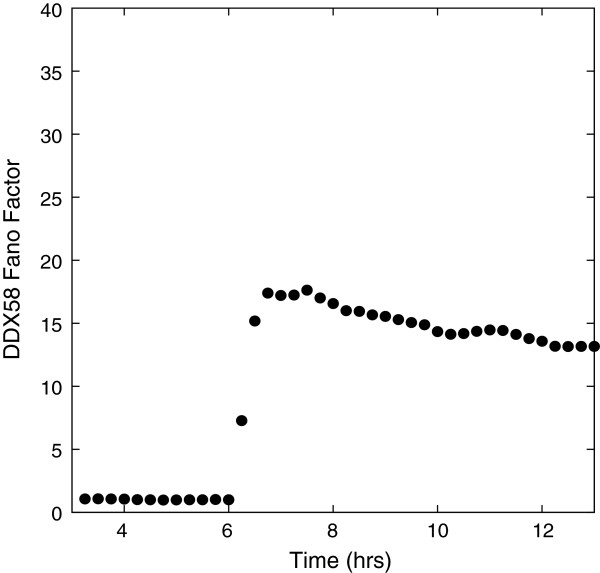
**DDX58 Fano factor as a function of time for cells with no viral infection but treated with of a uniform distribution of 1200 IFN-β proteins in each box.** The Fano factor is substantially lower than when spatially-induced noise occurs as in Figure 5.

To sharpen the point concerning the role of spatial heterogeneity and stochasticity of secreted cytokines, we complete a simple simulation in which we take all the cells to be uninfected and at 6 hours a uniform density of cytokines (1200 per cell) is introduced. This is the analog of pre-treatment in experiments except there is no subsequent viral infection. The Fano factor as a function of time is shown in Figure 7 and it shows a substantially smaller value of the Fano factor compared to Figure 5. At large times the Fano factor settles to the steady-state value for a bursting model that toggles between the enhanced and basal production rates. This provides a quantitative measure of the influence of the spatial heterogeneity of the cytokine sources due to viral infection and stochastic induction.

### Noise due to spatial heterogeneity depends on cell density and multiplicity of infection

**Figure 8:**
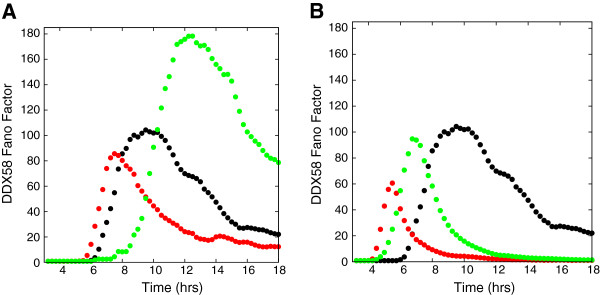
**The effect of the density of cells and multiplicity of infection on the extent and evolution of spatially heterogeneous noise as measured by the Fano Factor of the DDX58 distribution.** The effect of varying density is shown in **(A)**. We show results from a simulation with a quarter of the density used in the main text (green circles) and four times the density used in the text (red circles). The black circles denote a simulation without modification from the main text. In **(B)** the effect of varying the multiplicity of viral infection is shown. The green and red circles denote simulation with an MOI of .5 and 2.0 respectively. This simulation was carried out with the threshold for bound IFNAR to reach half-maximum production of DDX58 set to 10% of the number of bound receptors.

Experimentally, the spatial heterogeneity of the cytokine sources depends on the density and the multiplicity of infection. When these increase from the values used in our simulations, we expect the behavior of the Fano factor (used as a measure of the broadness of the distribution) to be closer to the result for the homogeneous excitation by cytokine stimulus only. When the density of cells is high, spatial homogenization occurs more rapidly and the noise may be difficult to observe. The variation of the Fano factor with time for different densities is displayed in Figure 8A. With a density reduced by a factor of four (green circles) from that used in the reported results, the induced noise is considerably enhanced and the Fano factor maximum occurs roughly two to three hours later. When the density is increased by a factor of four (red circles), the maximum in the Fano factor occurs slightly earlier and decays more rapidly.

We illustrate the effects of varying MOI (multiplicity of infection) in Figure 8B. Increasing MOI from 0.5 to 2.0 increases the fraction of infected cells that can express the IFN-β gene from 40% to 86%. This substantially reduces the initial spatial heterogeneity and attenuates the noise and the duration over which the induction is noisy, as seen in Figure 8B. This simulation is performed with a much lower value of the threshold fraction of bound IFNAR (10%) for both MOIs in comparison with the bulk of the simulations. These results establish the significant role played by cell density and MOI value in influencing the role of spatial heterogeneity and show how they affect the time scales and width of the noise distribution of interferon-induced genes.

### Induced intracellular noise depends on the threshold fraction of bound receptors and temporal fluctuations in cytokine secretion

**Figure 9:**
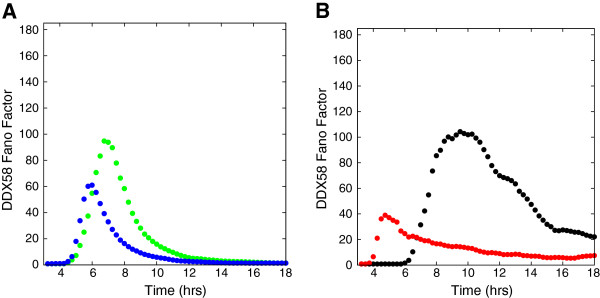
**The effect of different factors on the extent and evolution of spatially heterogeneous noise as measured by the Fano factor of the DDX58 distribution.** In both plots the black circles are for results of a simulation without modification from the main text. In **(A)** the threshold number of bound receptors at which the induced gene has half-maximum expression (see text) is varied. We display results when the threshold is set at 5% (blue circles) and 10% (green circles) of the total number of receptors. In **(B)** the effect of the temporal fluctuations in the secreted IFN-β is removed by doing a simulation with all the infected cells producing IFN-β at the same time. The results are shown as red circles. The Fano factor remains large due to spatial heterogeneity.

Under what conditions is the propagation of noise via extracellular signaling significant? The results of our model simulations in Figure 9 show how the mechanism we have proposed for spatial heterogeneity-induced noise depends on some important cell rate constants.

One of the key cell parameters that determine the effects under discussion is the value of the threshold fraction of bound receptors at which the enhancement of the induced gene *DDX58*’s production rate reaches half its maximum value. If the threshold value is low, the secreted cytokine density reaches a value sufficient to activate the JAK/STAT pathway in most cells rapidly attenuating cell-to-cell variability in induced gene expression. This is shown in Figure 9A where we use a threshold fraction of 5% and 10% in comparison to the 47.5% used elsewhere in our simulations. These changes not only decrease the magnitude of the Fano factor but also affect the time scales, the duration and the time of occurrence of noisy induction. Thus as the threshold fraction of bound receptors increases, the amount of noise increases; however, if the threshold fraction of bound receptors gets close to unity, noise in the induced genes can again be suppressed (by the time the cytokine density is large enough to bind enough receptors, it can become spatially homogeneous).

Suppressing temporal fluctuations in the production of cytokines reduces the noise in downstream genes but does not eliminate it, confirming the importance of the spatial source of the heterogeneity. There is remnant noise in the induced gene expression due to initial spatial fluctuations. The initial spatial inhomogeneity arises from the fact we use an MOI=0.5, as in the experiment, leading to about 40% of the cells being infected at random spatial locations. In addition to the initial spatial fluctuations, there is a contribution from the intrinsic noise in *DDX58* induction. The result for the Fano factor when the IFN-β proteins are secreted simultaneously contrasts in Figure 9B with the result of the main simulations where temporal stochasticity in IFN-β secretion plays an important role. The time scale over which the system becomes homogeneous is clearly considerably shortened and the maximum amplitude of the Fano factor decreases by a factor close to 3. Nevertheless, the induced gene expression level is still noisy because of the spatial heterogeneity. These results delineate how the threshold fraction of bound receptors and temporal fluctuations in cytokine secretion affect induced noise behavior.

## Discussion

We propose and investigate using numerical simulations a mechanism of the transmission of noise to cytokine-induced genes that arises due to spatial and temporal heterogeneity of the sources of cytokine production. The secreted cytokines diffuse and bind to the cellular receptors where signaling pathways are activated. The initial spatial heterogeneity of the cytokine induces downstream intracellular noise through extracellular signaling. We compare and validate our results with experimental single cell data for a population of dendritic cells infected by a virus.

Our model is a spatially-extended agent-based model of dendritic cells in two dimensions with intracellular processes representing the following: IFN-β induction, IFN-β export, extracellular diffusion of the cytokines, and binding of the cytokines to IFNAR. We include as an illustrative example a coarse-grained model of induced gene expression with *DDX58*. The spatial inhomogeneity of the cytokine leads to a broad distribution of bound receptors on the membranes of infected and uninfected cells. When this distribution spans the fraction of bound receptors needed for significant activation of the JAK/STAT pathway, considerable cell-to-cell variability occurs in the copy numbers of interferon-induced genes. This leads us to propose a general mechanism whereby temporal noise in cytokine production can cascade through extracellular diffusion to downstream-induced genes.

Another example of an interferon-induced gene (that is activated by means of the JAK/STAT pathway like *DDX58*) is MxA, which has been shown to exhibit anti-viral activity such as inhibiting viral multiplication of pandemic viruses like the 1918 flu [29-31]. Under the conditions stated, our mechanism would suggest that the MxA distribution should also be broad, which translates to a broad range of anti-viral activity across the cell population. This result is derived from a theoretical study of experimental results that depend on both the virus and cell type used. Zhao et al. [23] draw attention to the fact that even the percentage of activated cells depends on the cell line used. The work of Rand et al. [22] was done by infecting murine fibroblasts (transfected with plasmids) with NDV and the experiments of Zhao et al. used the Sendai virus as the infecting agent. On the other hand, our experiments were done on human DCs infected by NDV. The time scales involved in our measurements differ from those of Rand et al. Our experimental results are on times up to 10 hours post-infection, whereas the bulk of the changes on murine fibroblasts [22] occur between 12 and 20 hours. As to the interferon-induced gene expression, Rand et al. show (in their Supplementary Section) that after the addition of IFNβ to the cells the distribution of IRF-7 has a broad expression pattern with bimodality. In our experiments, however, the cells themselves secrete IFN-β as a result of viral infection. All these differences render difficult a direct comparison of our results with those of Zhao et al. and Rand et al. We note in passing that it is possible to obtain bimodal distributions for the interferon stimulated genes (Figure 6A hints at such a possibility) in this model; we check this by varying the rate constants for the transition rates between two possible states of the *DDX58* gene. This is consistent with the exact results obtained by us earlier for a simple bursting model [24].

In our simulations the source of the noise is the broad distribution of the times at which IFN-β induction commences in infected cells. We model this noise as resulting from the stochastic assembly of the enhanceosome and induction of *IFNB1* as observed in measurements of intrinsic noise [32]. Other sources of noise linked to cellular variability of the number of signaling molecules or other extrinsic noise emphasized by [23] would also lead to fluctuations in *IFNB1* induction times. The specific mechanism that leads to the initial fluctuations is not important; it is the temporally sporadic, spatially random cytokine secretion that drives downstream noise. Bi-allelic production of the IFN-β mRNA [21,32], which is not included in our model, will also increase the stochasticity since the two alleles can turn on at different times.

The distribution of DDX58 obtained from the simulations is in good agreement with experimental data. The broad distribution of DDX58 leads to a broad distribution of the product RIG-I protein (See Additional file 1: Figure S3). RIG-I is the key cellular sensor that initiates the anti-viral response of DCs and so the feedback loop through paracrine signaling primes other DCs over a broad time period and can include those DCs in which viral entry may occur late [33-36]. On the other hand we have shown that autocrine signaling dominates at early times (4 – 6 hours post-infection), which enhances the impact of those cells that respond early to viral intrusion.

We demonstrated that at early times (4 – 6 hours post-infection) autocrine signaling is dominant and enhances the cell-to-cell variability in IFN-β due to stochastic induction. At intermediate times (6 – 8 hours post-infection) the paracrine signaling produces large variability across the cell population in the interferon-stimulated genes. At later times (> 8 hours post-infection) paracrine signaling dominates and begins to quench the cell-to-cell variability of the interferon-stimulated genes.

We have identified, motivated by our measurements, how noise can be amplified by diffusive signaling in the transient state following viral infection of human dendritic cells and investigated the conditions under which it can occur. Though there has been work on modeling intercellular communication, none of these studies focus on the effects of noise in the transient state and how intracellular, intrinsic, stochasticity leads to further cell-to-cell variability, via intercellular communication. In the context of quorum sensing, coupled stochastic differential equations have been used [37,38] to investigate in the steady state the effect of noise on gene expression in bacteria. Results of multiscale, stochastic modeling of various phenomena in immunology have been reported, for example, in host-pathogen interactions [39], the modeling of lymph nodes [40], and the resolution of inflammation by leukocytes [41]. In a recent study, the influence of cytokines following tuberculosis infection on the granuloma environment of lung tissue has been studied with agent-based modeling [42]. Importantly, all of these studies involve different time or length scales from the scales presented here.

## Conclusions

In this paper we propose a general mechanism by which intracellular noise in protein numbers can be transmitted in the transient state via extracellular communication to induced downstream genes in other cells. The specific process by which the temporal noise in the proteins is produced is not important. We illustrated our general mechanism in the context of the experimentally studied system of dendritic cells exposed to a viral antagonist.

We have identified external factors that can significantly affect the level of noise and relevant time scales - in particular, the density of cells (an important element in interpreting experiments) and the level of infection as measured by the fraction of infected cells. Among the intrinsic factors that impact the propagation of noise and response of a cell population, an important one is the threshold number of bound receptors. This number can, therefore, affect the overall noise in the anti-viral response of the cell population.

The mechanism of cellular stochasticity of gene induction, which we have investigated by means of an ABM with a systems biology approach, is not limited to the particular interferon-stimulated gene we studied; a broad distribution across cells of copy number is to be expected for other interferon-stimulated genes. This can lead to functional consequences for cell-to-cell variability in the range of anti-viral states of a population of cells and is a subject for future study.

## Methods

All the intracellular interactions used in the simulations and the corresponding rate constants are included in Additional file 1: Tables S1, S2, S3, S4, and S5.

We simulated the stochastic intracellular processes of both infected and uninfected DCs using the standard Gillespie algorithm [43]. The method used to simulate the extracellular diffusion of IFN-β is described in detail in [19]. The code was written in C++ and the computations were performed on a PC.

We modeled the system *in silico* on a two dimensional lattice of 40 × 40 squares that represents the medium. The size of the squares is chosen to be slightly larger than the diameter of a DC thus allowing at most one cell per square. The system contained 210 DCs uniformly randomly distributed with 40% of the DCs infected corresponding to an MOI of 0.5. The average diameter of a DC is 30 μm. In the simulation we used a density of roughly 5 × 10^6^ cells mL^-1^. The simulation volume can be viewed as a region of space with a thickness chosen to be the diameter of a DC and a cross-sectional area of (1200 μm)^2^.

The parameters for the intercellular modeling were based on experimental data and previous *in silico* simulations [18]. The experimental value for the diffusion coefficient for the cytokine IFN-β is approximately 10 μm^2^ s^-1^. Given the diffusion coefficient and the dimensionality of a lattice box we determined the simulation diffusion time step of 11.25 s for the IFN-β to diffuse to an adjacent square with 50% probability (See Additional file 1 for a derivation of the simulation diffusion time step).

We describe the intracellular part of the simulation next. The binding and unbinding of IFN-β to the IFNAR reactions are included in the intracellular part of the algorithm. This is justified because only the IFN-β proteins inside a lattice box containing a DC have a non-zero probability of being bound to a free IFNAR on the cell surface. The rate constants for the binding and unbinding of IFN-β to the IFNAR are chosen based on the literature [44].

The induction of IFN-β is described by an extension of an intracellular model of IFN-β mRNA induction developed earlier to explain experimentally observed power-law behavior in IFN-β mRNA enhanceosome [32]. It has been shown experimentally [45,46] that the assembly of the enhanceosome is promoted by HMGI, an architectural protein; NFκB is detected initially at the promoter with an IRF and ATF-2 recruited, later followed by the arrival of IRF-3 or IRF-7. We model the enhanceosome with the sequential cooperative binding of four proteins. As in the earlier model, the cascade of steps required for the assembly of the pre-initiation complex is represented by a single step that takes the assembled enhanceosome to a transcribing state. This gives rise to bursting-type kinetics [9,24,25,47] that leads to good agreement with the experimentally observed power-law distributions of the IFN-β mRNA copy number [32].

We do not explicitly model the stochastic activation of RIG-I and the transcription factors involved in the enhanceosome assembly to keep the model tractable; however, we modify the forward rates for the assembly of the enhanceosome to be a function of RIG-I number. This represents the complex set of reactions that ensue following viral detection by RIG-I in the cytosol. The transcription rate of IFN-β mRNA was modified to be a function of bound IFNAR. This was done to simulate the increased production of IFN-β mRNA through signal transduction by means of the exchange of IRF-3 with the induced IRF-7 [48]. The rate constants for the up regulation of IFN-β mRNA’s transcription rate via the exchange of IRF-3 with induced IRF-7 were chosen to match the experimental IFN-β mRNA distribution [18].

The IFN-β secretion was modeled directly as translation of IFN-β mRNA and export because the transport of the IFN-β is deemed to be rapid with a rate of secretion chosen based on the [18] paper. We emphasize that the intracellular reactions involving IFN-β occur only in infected cells.

We use a more detailed model of *DDX58* gene induction than used previously: the gene is assumed to have two states, a basal (low) production state in the absence of the activated JAK/STAT pathway and an enhanced production state after activation. The transition rate of the *DDX58* gene from a low to a high production state was modeled in a coarse-grained way as a function of the number of bound IFNARs; this determines the level of signal transduction of the JAK/STAT pathway that in turn enhances the production of DDX58. The chemical reactions for DDX58 and rate constants are found in the Additional file 1: Tables S2, S3, and S4. The rate constant for DDX58 degradation was chosen to be the same as in earlier work [18]. The production rates in the two states of the gene and the transition rate between them were chosen to match the average number of DDX58 at six and eleven hours observed experimentally. The RIG-I production was modeled as translation of DDX58 with the same rate constants as used earlier [18].

### Sensitivity analysis

It is computationally infeasible to do a systematic variation of all the rate constants and parameters in the model. It is, however, important to understand the effects of changing specific model ingredients. Since we have a clear mechanistic understanding of the proposed mechanism for explaining the experimentally observed broad distribution of DDX58 in human DCs with NDV infection, we vary the rate constants for the key reactions that affect the noise by roughly an order of magnitude: from 1/3 or 1/4 of the value used in the reported results to 3 or 4 times the value, in order to understand the robustness of the results and identify the rate constants to which the results are most sensitive. We summarize the results here and provide the relevant figures in the Additional file 1.

We modify the IFN-β mRNA induction model to include the two-stage induction process reported in the literature. It is known [48] that IRF-7 is the master regulator and is more efficient in promoting IFN-β transcription but its constitutive production is negligible in m-DCs. It is induced through the JAK/STAT pathway by the secreted IFN-β protein. We have modeled this two-stage process by using a lower production rate of mRNA initially in the presence of the constitutively expressed IRF-3 and a higher rate after a fraction of the receptors (IFNAR) are bound. Choosing rate constants that fit the observed IFN-β data leaves all of our main results for the induced gene unaffected.

In the model we vary the rate at which the enhanceosome (once it is formed) is activated and deactivated; this changes the power-law distribution observed experimentally [32] and eliminates it entirely in one case. The activation rate constant for the IFN-β gene controls the location of the DDX58 Fano factor peak: the peak time occurs later by as much as 90 minutes as the activation rate is decreased (see Additional file 1: Figure S5). The activation rate constant for the IFN-β gene after the enhanceosome is formed also controls how rapidly the infected DCs become active. A three-fold increase or decrease varies the percent of activated infected DCs by up to approximately 30% while the noise in the interferon-induced genes remains significant.

Another important parameter is the binding rate of IFN-β protein to IFNAR. A four-fold increase in the binding rate decreases the time of occurrence of the Fano factor peak by about an hour, while a four-fold decrease delays the peak position by more than 2 hours. The effect on the ratio of autocrine to paracrine signaling is not large, although autocrine signaling is somewhat enhanced as the binding rate increases (see Additional file 1: Figure S6).

We check that our results are insensitive to the initial conditions, for example, the precise spatial distribution of infected cells. We test the importance of statistical fluctuations (the number of cells is 210 in the experiments and for the results reported here) by simulating 1050 cells, keeping the same density of cells. Within the expected level of fluctuations the results remain the same. Changing the size of the boxes by a few percent (still keeping at most one DC per box) left the results substantially unaltered. In summary, when the spatio-temporal production of the cytokine is heterogeneous, the mRNA distribution of the induced genes across cells can be broad and we have studied the effect of different factors on this phenomenon.

## Abbreviations

NDV: Newcastle disease virus; DCs: Dendritic cells; IFNAR: Interferon-α/β receptor; ISRE: Interferon-stimulated response element; MOI: Multiplicity of infection.

## Competing interest

The authors declared that they have no competing interest.

## Authors’ contributions

FH and CJ conceived the project. CJ, GN, and OPT contributed to the algorithm development. OPT performed the simulations. FH, CJ, SCS, and OPT wrote the paper. All authors read and approved the final manuscript.

## Supplementary Material

Additional file 1 Supplementary information. Click here for file
