# Supplementary material for: Noise propagation through extracellular signaling leads to fluctuations in gene expression
Source: BMC Syst Biol. 2013 Sep 25;7:94. doi: 10.1186/1752-0509-7-94 (PMC3906959; doi:10.1186/1752-0509-7-94)
Supplement: Additional file 1 — Supplementary information. [file 1752-0509-7-94-S1.docx]

**Additional file 1**

Omar P. Tabbaa^1^, German Nudelman^2^, Stuart C. Sealfon,^2,3^ Fernand Hayot^2,3^  and C. Jayaprakash^1,*^

^1^Department of Physics, Ohio State University, Columbus, 43210, United States of America

^2^Department of Neurology, Mount Sinai School of Medicine, New York, 10029, United States of America
^3^Center for Translational Systems Biology, Mount Sinai School of Medicine, New York, 10029, United States of America

* Corresponding author

Table of Contents

Supplementary Figures 2

Supplementary Text 8

Supplementary Tables 10

References 15

# Supplementary Figures

Figure S1: Plot of S(t), a measure of the relative contributions of autocrine and paracrine signaling, as a function of time. Let B*(t) and B(t) denote the average number of bound IFNAR on cells with and without IFN-β mRNA at time t respectively. The former is determined by both autocrine and paracrine signaling while the latter arises from paracrine signaling only. As a quantitative measure of their relative importance we have defined S(t) = (B*(t) - B(t))/(B*(t) +B(t)). When the signaling is all autocrine (B(t)=0) S(t) equals 1, for equal contributions from autocrine and paracrine signaling (B*(t)=2B(t)) S(t) equals 1/3, and for purely paracrine signaling (B*(t)=B(t)) S(t) equals 0. We see that after about 6 hours paracrine signaling dominates.

**A**

**B**

**C**

Figure S2: Spatial distribution of bound IFNAR receptors in all cells. The distributions at (A) 6 hours (B) 9 hours and (C) 11 hours post infection are displayed. The colors indicate the range of IFNAR numbers: black denotes bound IFNAR numbers fewer than 250 per cell, green a number between 250 and 500, orange a number greater than 500. At early times (less than 6 hours) less than 1% of cells display bound receptor numbers fewer than the threshold and the majority of cells that have greater than the threshold are IFN-β producing (see Figure 4 in main text) reflecting the dominate role of autocrine signaling. The number of cells with bound receptor numbers larger than the threshold values progressively increases to a homogenous state of all cells having greater than the threshold number of bound receptors as the paracrine signaling becomes the dominate type of signaling at later times (approximately 11 hours).

**A**

**B**

Figure S3: The RIG-I copy number distribution at (A) 6 hours and (B) 11 hours post infection. The long (approximately 3-hour) degradation rate of the RIG-I protein allows accumulation in cells in which the gene is induced early. This leads to a long tail (RIG-I number greater than 3000) at 11 hours shown in the inset that accounts for a sixth of the cells.

**A**

**B**

Figure S4: (A) Time averaged DDX58 transcription rate as a function of the number of bound IFNAR. This is obtained by deriving an effective deterministic equation for DDX58 transcription from the reactions in Supplementary Table S2. (B) Time evolution of the fraction of cells with bound IFNAR between 200 and 600 (the threshold value for half-maximum rate of DDX58 gene induction is 475). The green points correspond to cells with IFN-β mRNA and black points to those without. The fraction of cells with substantial bound IFNAR increases earlier for cells with IFN-β mRNA in contrast to cells without, reflecting the importance of autocrine signaling at early times between 5.5 and 6.5 hours. The fraction of cells with a bound IFNAR number between 200 and 600 decreases beyond 9 hours because the bound IFNAR number exceeds 600.

Figure S5: Dependence of the Fano factor of the induced gene (DDX58) on the activation rate of the IFNB1 gene. The black points denote the temporal variation of the Fano factor with the activation rate constant, c_f_ in Supplementary Table S4, set equal to the original value used in the main text. The effect of increasing the activation rate three-fold from the original value (red points) and decreasing it three-fold (green points) is asymmetric. The change is minimal for the increase while the three-fold decrease delays the time at which the maximum occurs by more than 90 minutes.

Figure S6: The effect of the variation of the binding rate of IFN-β binding to a free IFNAR on the time variation of the Fano factor of the induced gene. The behavior when the binding rate, k_b_ in Supplementary Table S4, is equal to the original value used in the main text is shown as black points. A four-fold increase in the binding rate with respect to the original value decreases the maximum Fano factor slightly but speeds up the approach to the steady-state value. When the binding rate is decreased four-fold the peak value is delayed but increased and the decay is considerably slower.

#

Figure S7: The combined distributions of Figure 4 (panels A-C) of bound interferon receptors (IFNAR) for cells with and without IFN-β mRNA. For cells containing IFN-β mRNA (black border) and for cells with no IFN-β mRNA (blue border) plotted at 6 hours. The larger fraction of bound receptors in cells with IFN-β mRNA above 200 in comparison to cells without IFN-β mRNA (at 6 hours) indicates the importance of autocrine signaling at early times (4 – 6 hours post infection). The distribution of bound IFNAR at 11 hours post infection is displayed for cells with (red border) and for cells without (green border) IFN-β mRNA.

# Supplementary Text

Text S1: **Analytic Calculation for the Diffusion Time Step**

We provide a theoretical derivation of the numerical value of the time step, $\tau$, for the diffusion of IFN-$\beta$ used in the simulations. The probability of the protein being at the position $(x,y)$ on the lattice at time $t+\tau$ obeys the discrete diffusion equation

$P\left( x,y,t+\tau\right)= \left( 1-p \right)P\left( x,y,t \right)+ \frac{p}{4} \left[ P\left( x+a,y,t \right)+P\left( x-a,y,t \right)+P\left( x,y+a,t \right)+P\left( x,y-a,t \right) \right].$ (1)

This equation states that in a time $\tau$ the protein stays in the original position with probability $(1-p)$ chosen to be $1/2$ or moves one step to the four nearest points with probability $p/4$ [1]. The spatial distance moved is the size of the box used in the simulations, $30 \mu m$. We will obtain an expression for the effective diffusion constant from this equation and equate it to the value $10 \mu m^{2}/s$ for the IFN-$\beta$ protein in the supernatant [2]. This allows us to determine $\tau$. The expression for the diffusion constant is obtained by applying a Taylor series expansion in powers of $a$ to Equation (1) and retaining the leading powers of space and time derivatives. We obtain

$\frac{\partial P}{\partial t} = \frac{p a^{2}}{4\tau} \nabla^{2} P .$ (2)

This is the standard form of the diffusion equation in continuous space-time and allows one to immediately identify the diffusion constant $D$ to be $\frac{p a^{2}}{4\tau}$. Using the values given above we find $\tau= 11.25s$ the value used in our simulations.

#

# Supplementary Tables

Table S1:

| Molecule | Abbreviation |
| --- | --- |
| The empty IFN-$\beta$ gene | D |
| The first stage of the assembled enhanceosome | Ds1 |
| The second stage of the assembled enhanceosome | Ds2 |
| The third stage of the assembled enhanceosome | Ds3 |
| The enhanceosome without the activators | Ds4 |
| The activated IFN-$\beta$ gene | Ds4* |
| RIG-I mRNA | DDX58 |
| RIG-I protein | RIG-I |
| IFN-β mRNA | IFN$\beta$m |
| IFN-β protein | IFN$\beta$ |
| Free interferon receptor | IFNAR |
| Bound interferon receptor | IFNAR* |
| DDX58 gene in the basal transcription state | G |
| DDX58 gene in the enhanced transcription state | G* |

Table S1: The above table lists the molecule types used in our model and the respective abbreviations used in the text and the Tables.

Table S2:

| Intracellular Reactions | Description of Reaction | Propensities |
| --- | --- | --- |
| $D \overset{k_{1}^{\mathrm{eff}}}{\underset{k_{-1}}{}} Ds1$ | First step in enhanceosome assembly | Forward: $k_{1}^{\mathrm{eff}} D$  Reverse: $k_{-1} Ds1$ |
| $Ds1 \overset{k_{1}^{\mathrm{eff}}}{\underset{k_{-1}}{}} Ds2$ | Second step in enhanceosome assembly | Forward: $k_{1}^{\mathrm{eff}} Ds1$  Reverse: $k_{-1} Ds2$ |
| $Ds2 \overset{k_{1}^{\mathrm{eff}}}{\underset{k_{-1}}{}} Ds3$ | Third step in enhanceosome assembly | Forward: $k_{1}^{\mathrm{eff}} Ds2$  Reverse: $k_{-1} Ds3$ |
| $Ds3 \overset{k_{1}^{\mathrm{eff}}}{\underset{\frac{k_{-1}}{4}}{}} Ds4$ | Fourth step in enhanceosome assembly | Forward: $k_{1}^{\mathrm{eff}} Ds3$  Reverse: $\frac{k_{-1}}{4} Ds4$ |
| $Ds4 \overset{c_{f}}{\underset{c_{b}}{}}\mathrm{Ds}4^{*}$ | Activation/deactivation of IFN-$\beta$ gene | Forward: $c_{f} Ds4$  Reverse: $c_{b} \mathrm{Ds}4^{*}$ |
| $G\underset{\to}{k_{\mathrm{DDX}}^{\mathrm{low}}}G+DDX58$ | Basal transcription of DDX58 | $k_{\mathrm{DDX}}^{\mathrm{low}} G$ |
| $G^{*}\underset{\to}{k_{\mathrm{DDX}}^{\mathrm{high}}}G^{*}+DDX58$ | Enhanced transcription of DDX58 | $k_{\mathrm{DDX}}^{\mathrm{high}} G^{*}$ |
| $DDX58\underset{\to}{\delta_{\mathrm{DDX}}}\phi$ | Degradation of DDX58 | $\delta_{\mathrm{DDX}} DDX58$ |
| $G \overset{c_{b}(\beta\mathrm{IFNAR}^{*})^{2}}{\underset{c_{b}}{}} G^{*}$ | Activation/deactivation of DDX58 gene | Forward: $c_{b}(\beta\mathrm{IFNAR}^{*})^{2} G$  Reverse: $c_{b} G^{*}$ |
| $DDX58\underset{\to}{k_{\mathrm{RIG}\text{-}I}}DDX58+RIG\text{-}I$ | Translation of RIG-I | $k_{\mathrm{RIG}\text{-}I} DDX58$ |
| $\mathrm{RIG}\text{-}I\underset{\to}{\delta_{\mathrm{RIG}\text{-}I}}\phi$ | Degradation of RIG-I | $\delta_{\mathrm{RIG}\text{-}I} \mathrm{RIG}\text{-}I$ |
| $\mathrm{Ds}4^{*}\underset{\to}{k_{\mathrm{IFN}}^{\mathrm{eff}}}\mathrm{Ds}4^{*}+IFN\beta m$ | Transcription of IFN-$\beta$ mRNA | $k_{\mathrm{IFN}}^{\mathrm{eff}} \mathrm{Ds}4^{*}$ |
| $IFN\beta m\underset{\to}{\delta_{\mathrm{IFN}}}\phi$ | Degradation of IFN-$\beta$ mRNA | $\delta_{\mathrm{IFN}} IFN\beta m$ |
| $IFN\beta m\underset{\to}{k_{s}}IFN\beta$ | Secretion of IFN-$\beta$ | $k_{s} IFN\beta m$ |
| $IFN\beta+ IFNAR \overset{k_{b}}{\underset{k_{\mathrm{ub}}}{}}\mathrm{IFNA}R^{*}$ | Binding and Unbinding of IFN-$\beta$ to the interferon receptors | Forward: $k_{b}IFN\beta IFNAR$  Reverse: $k_{\mathrm{ub}} \mathrm{IFNA}R^{*}$ |

Table S2: The above table gives the reactions, reaction descriptions, and the propensites that determine the probability for the reaction to occur in our simulations.

Table S3:

| Effective Rate Constants | Definition |
| --- | --- |
| $k_{1}^{\mathrm{eff}}$  (forward rate of enhanceosome assembly) | $\frac{k_{1}}{10}+\frac{{2 k}_{1}}{10}\frac{(\gamma RIG{\text{-}I)}^{3}}{1+(\gamma RIG\text{-}I)^{3}}$ |
| $k_{\mathrm{DDX}}^{\mathrm{high}}$  (high transcription rate of DDX58 production) | $k_{\mathrm{DDX}}^{\mathrm{low}}(1+\eta)$ |
| $k_{\mathrm{IFN}}^{\mathrm{eff}}$  (rate of IFNβ production) | $\frac{3k_{\mathrm{IFN}}}{4}+\frac{k_{\mathrm{IFN}}}{2}\frac{(\beta\mathrm{IFNAR}^{*})^{4}}{1+(\beta\mathrm{IFNAR}^{*})^{4}}$ |

Table S3: The above table gives the effective rate constants for the forward rate of enhanceosme assembly ($k_{1}^{\mathrm{eff}}$), the high transcription rate of DDX58 production ($k_{\mathrm{DDX}}^{\mathrm{high}}$), and the rate of IFN-$\beta$ production ($k_{\mathrm{IFN}}^{\mathrm{eff}}$). RIG-I and IFNAR* denote the corresponding number of molecules per cell. See the Methods section of the main text for an explanation of the effective rate constants.

Table S4:

| Description of rate constant | Variable name | Numerical Value |
| --- | --- | --- |
| Rate constant for a enhanceosome component binding | $k_{1}$ | $0.00136s^{-1}$ |
| Rate constant for a enhanceosome component unbinding | $k_{-1}$ | $5.46*10^{-3}s^{-1}$ |
| RIG-I number when the enhancement component of the rate constant for a enhanceosome component binding is half maximum | $\frac{1}{\gamma}$ | 5750 |
| Basal transcription rate of DDX58 | $k_{\mathrm{DDX}}^{\mathrm{low}}$ | $0.005s^{-1}$ |
| Number of bound IFNAR at which the DDX58 transcription rate is half maximum | $\frac{1}{\beta}$ | $475$ |
| DDX58 degradation rate constant | $\delta_{\mathrm{DDX}}$ | $10^{-4}s^{-1}$ |
| Activation rate of the IFNB1 gene | $c_{f}$ | $1.5*10^{-4}s^{-1}$ |
| Deactivation rate of the IFNB1 gene | $c_{b}$ | $3*10^{-5}s^{-1}$ |
| RIG-I translation rate constant | $k_{\mathrm{RIG}\text{-}I}$ | $\frac{1}{262.33}s^{-1}$ |
| RIG-I degradation rate constant | $\delta_{\mathrm{RIG}\text{-}I}$ | $10^{-4}s^{-1}$ |
| Maximum transcription rate of IFN-$\beta$ mRNA | $k_{\mathrm{IFN}}$ | $\frac{1}{7.5}s^{-1}$ |
| Rate constant for the binding of IFN-$\beta$ to a free IFNAR | $k_{b}$ | $10^{-2}nM^{-1}s^{-1}$ |
| Rate constant for the unbinding of IFN-$\beta$ from a bound IFNAR | $k_{\mathrm{ub}}$ | $10^{-3}s^{-1}$ |
| IFN-$\beta$ secretion rate constant | $k_{s}$ | $\frac{1}{90}s^{-1}$ |
| Enhancement factor for DDX58 transcription | $\eta$ | $20$ |
| IFN-$\beta$ mRNA degradation rate constant | $\delta_{\mathrm{IFN}}$ | $10^{-4}s^{-1}$ |

Table S4: The above table gives the rate constants and parameters used in our simulations. These numbers are based on estimates in previously published work and experimental data [2-7]. See the Methods section of the main text for more details.

Table S5:

| Molecule | Initial Condition |
| --- | --- |
| DDX58 | Poisson distribution with a mean of 50 |
| IFN$\beta$m | 0 |
| IFNAR | 1000 |
| IFNAR* | 0 |
| D | 1 |
| Ds1 | 0 |
| Ds2 | 0 |
| Ds3 | 0 |
| Ds4 | 0 |
| Ds4* | 0 |
| RIG-I | 1906 |
| IFN$\beta$ | 0 |
| G | 1 |
| G* | 0 |
| Density of Cells | $5*10^{6} cells/mL$ |
| Multiplicity of Infection | 0.5 |
| Volume of a Cell | $1.4*10^{4} {\mu m}^{3}$ |

Table S5: The above table gives the initial number of molecules per dendritic cell, the density of cells used in the simulations, the multiplicity of infection, and the volume of each cell. These numbers are based on estimates in previously published work and experimental data [2-7]. See the Methods section of the main text for more details.

# References

1. Gardiner CW (2004) The Random Walk in One Dimension. In *Handbook of stochastic methods* (pp. 70-73). Berlin: Springer.
2. Shimoni Y, Nudelman G, Hayot F, and Sealfon SC: **Multi-scale stochastic simulation of diffusion-coupled agents and its application to cell culture simulation.** *PLoS One* 2011, **6**:e29298-e29298
3. Hu J, Nudelman G, Shimoni Y, Kumar M, Ding Y, López C, Hayot F, Wetmur JG, and Sealfon SC: **Role of Cell-to-Cell Variability in Activating a Positive Feedback Antiviral Response in Human Dendritic Cells.** *PLoS One* 2011, **6**: 1661-1664.
4. Coppey M, Berezhkovskii AM, Sealfon SC, and Shvartsman SY: **Time and length scales of autocrine signals in three dimensions.** *Biophys J* 2007, **93**: 1917-1922.
5. Hu J, Iyer-Biswas S, Sealfon SC, Wetmur J, Jayaprakash C, and Hayot F: **Power-laws in interferon-b mRNA distribution in virus-infected dendritic cells.** *Biophys J* 2009, **97**: 1984-1989.
6. Honda K, Yanai H, Negishi H, Asagiri M, Sato M, Mizutani T, Shimada N, Ohba Y, Takaoka A, Yoshida N, and Taniguchi T: **IRF-7 is the master regulator of type-I interferon-dependent immune responses.** *Nature* 2005, **434**: 772-777.Munshi N, Agalioti T, Lomvardas S, Merika M, Chen G, and Thanos D: **Coordination of a transcriptional switch by HMGI(Y) acetylation.** *Science* 2001, **293**:1133-1136.
7. Panne D, Maniatis T, and Harrison SC: **An atomic model of the Interferon-β enhanceosome.** *Cell* 2007, **129:** 1111-1123.
